# ANCA vasculitis: A manifestation of Post-Covid-19 Syndrome

**DOI:** 10.1016/j.rmcr.2021.101549

**Published:** 2021-11-11

**Authors:** Desiree Morris, Kushal Patel, Osman Rahimi, Omar Sanyurah, Alfredo Iardino, Nazia Khan

**Affiliations:** a Kirk Kerkorian School of Medicine at UNLV, USA; b Department of Internal Medicine, Kirk Kerkorian School of Medicine at UNLV, USA; c Department of Internal Medicine, Division of Pulmonary Diseases and Critical Care Medicine, Kirk Kerkorian School of Medicine at UNLV, USA

**Keywords:** COVID-19, ANCA-Associated vasculitis, Respiratory medicine, Renal medicine

## Abstract

The SARS-CoV-2 infection has been found to present with different degrees of response and variable levels of inflammation. Patients who have recovered from the initial infection can develop long-term symptomatology. We present a unique case of a middle aged-healthy man who developed complications of ANCA-associated vasculitis after recovering from a mild COVID-19 infection.

A previously healthy 53-year-old male presented with hemoptysis and acute renal failure. One month prior, the patient tested positive for COVID-19; not requiring hospitalization. Physical exam findings included bilateral lower extremity petechiae. CT Chest showed bilateral diffuse patchy lung consolidations with cavitary lesions with urinalysis revealing erythrocytes, +1 protein. Hemodialysis and workup for pulmonary-renal syndromes were initiated.

Infectious workup results included: negative COVID-19, negative MTB-PCR, respiratory culture revealing yeast. Additional workup revealed; elevated CRP, D-Dimer, and Fibrinogen. Notably, the patient had; decreased C3 and C4 levels; negative Anti-GBM antibody; negative Anti-streptolysin-O; and positive ANCA assay, Proteinase antibody, and mildly positive Myeloperoxidase antibody.

Worsening coagulopathy and atrophic kidneys delayed renal biopsy for definitive diagnosis. The patient's respiratory status acutely worsened during hemodialysis with imaging showing markedly increased pulmonary infiltrates. Upon urgent intubation, active frank red bleeding was noted, and the patient sustained 2 cardiac arrests with eventual expiration.

Much is to be learned from the Novel SARS-CoV-2 virus and suspected complications. This case highlights a unique complication of COVID-19 leading to a possible AAV and the importance of keeping a broad differential when treating patients who have recovered from the initial infection.

## Highlights

• COVID-19 renal complications.
• ANCA-associated vasculitis after COVID-19.
• Acute renal failure after resolution of COVID-19.

## 1 Introduction

The emergence of the novel coronavirus or severe acute respiratory syndrome virus (SARS-CoV-2) and its associated human-to-human transmission played a major role in its subsequent outbreak leading to a public health crisis [1,2]. The severity of symptomatic infection of SARS-CoV-2 lies on a spectrum ranging from mild to severe illness with those with advanced age or previous underlying medical comorbidities being at increased risk for the latter. Globally, over 81 million cases of SARS-CoV-2 have been reported [3]. Based on cases with known symptoms reported to the CDC, the most common associated symptoms are cough, fever, myalgia, headache, dyspnea, sore throat, diarrhea, nausea/vomiting, loss of smell or taste, abdominal pain, and rhinorrhea, respectively [4]. Symptom severity can progress over the course of one week leading to dyspnea and hospital admission [5]. Several complications of SARS-CoV-2 infection have been described; including, acute respiratory distress syndrome marked by dyspnea, cardiomyopathy, thromboembolic complications, neurologic complications, and in some cases, inflammatory complications marked by elevated inflammatory markers. Patients who have recovered from the initial infection can develop long-term symptomatology and chronic conditions defined as post-acute COVID-19 syndrome [6].

We report a unique case describing a previously healthy middle-aged Hispanic male who developed complications of ANCA-associated vasculitis after recovering from a mild COVID-19 infection.

## 2 Case presentation

A previously healthy 53-year-old Hispanic male presented to the Emergency Department with a cough and non-massive hemoptysis. The patient had recently been diagnosed with COVID-19 one month prior and did not require hospitalization. The patient reported recovery but had a recurrent cough, hemoptysis, and centralized chest pain that started two weeks prior with a progressively worsening cough. Fatigue, generalized muscle pain, and loss of appetite were also noted. The patient denied fever, shortness of breath, nausea, vomiting, and/or diarrhea at the time of presentation. The patient denied any history of prior kidney disease, diabetes mellitus, exposure to Tuberculosis, history of drug abuse, tobacco, and alcohol intake. On physical examination, the patient was tachycardic without signs of acute distress, the physical exam was unremarkable. Initial laboratory results are shown in Table 1.

**Table 1:** Admission laboratory data.

Study	Value	Normal Values
White blood cell count, x 103/μL	25.56	4.8–10.8
Hemoglobin, g/dL	14.7	12–16
Platelets, × 103/μL	249	130–400
Glucose, mg/dL	132	70–110
BUN, mg/dL	107	7–20
Creatinine, mg/dL	5.8	0.6–1.2
Calcium, mg/dL	8	8.4–10.2
Sodium (Na), mmol/L	128	136–145
Potassium (K), mmol/L	5.5	3.5–5.1
Chloride (Cl), mmol/L	98	98–110
Albumin, mmol/L	2.9	3.5–5.0
Aspartate aminotransferase (AST), IU/L	60	3–38
Alanine aminotransferase (ALT), U/L	77	≤49
Alkaline Phosphatase, U/L	132	41–107

**Fig. 1:**
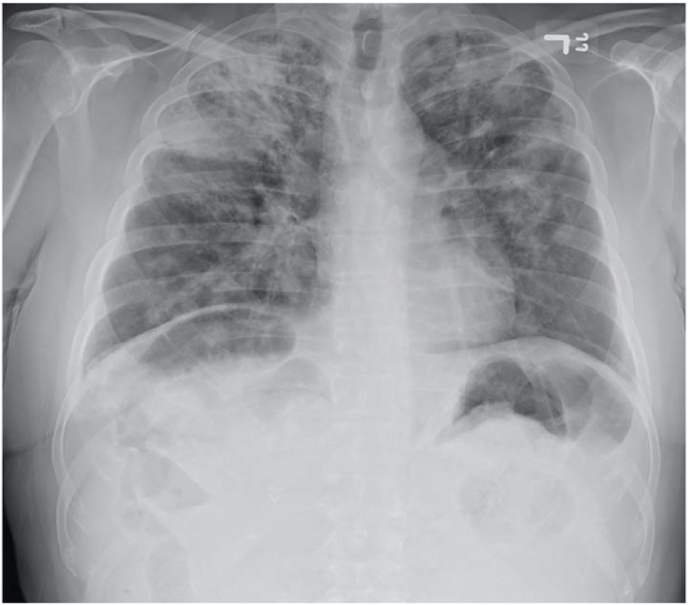
Chest X-ray. Chest X-ray shows bilateral scattered infiltrates, denser with partial consolidation in the right upper lobe bordering the minor fissure.

An electrocardiogram showed sinus tachycardia with no ST elevations or depressions. Initial Chest X-Ray revealed bilaterally scattered infiltrates that were denser with partial consolidation in the right upper lobe bordering the minor fissure (Fig. 1).

**Fig. 2:**
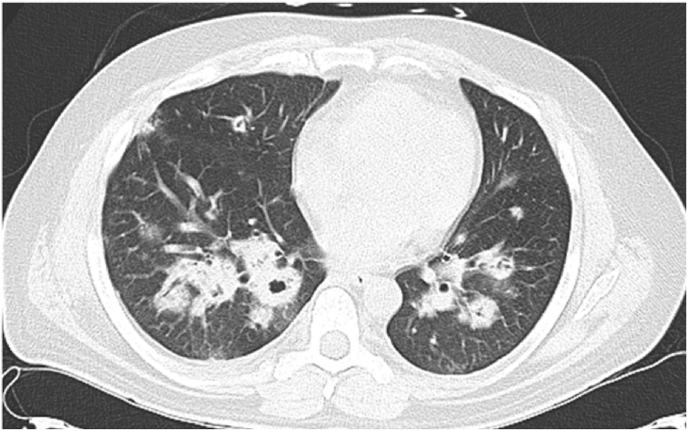
Computed Tomography Chest. CT Chest shows multiple patchy consolidations throughout both lungs, some of which are cavitating and are located primarily at the periphery.

CT of the chest with no contrast showed multiple patchy consolidations throughout both lungs of which some are cavitating and are located primarily at the periphery (Fig. 2).

Findings were likely suggestive of multifocal pneumonia.

The patient's blood, urine, and sputum cultures were taken. Urine analysis, protein/creatinine ratio, FeNa, urine osmolality, and SARS-CoV-2 real-time polymerase chain reaction (RT-PCR) were ordered. Additionally, hepatitis and human immunodeficiency (HIV) panels were ordered. The patient received antibiotic therapy targeting community-acquired pneumonia with Azithromycin 500 mg IV and Ceftriaxone 2g IV, and Sodium bicarbonate drip at 125 cc/hr and emergent hemodialysis per nephrology recommendation. Pulmonology was also consulted on admission.

Infectious workup results in the setting of acute kidney injury with pulmonary-renal disease are shown in Table 2.

**Table 2:** Fungal, bacterial, and viral serology.

Study	Value	Normal Values
Respiratory culture	yeast	no growth
Mycobacterium Tuberculosis PCR	negative	negative
Coccidioides Antibody CF titer	negative	negative
Aspergillus antigen	negative	negative
Fungitell (1–3)-B-D-Glucan	negative	negative
Blastomyces antibodies	negative	negative
Acid Fast Bacteria Culture	negative	negative
Hepatitis Panel	negative	negative
HIV Panel	negative	negative

Additional inflammatory and autoimmune workup revealed; CRP >200 mg/L, D-Dimer 6.41 mg/L, Fibrinogen 561 mg/dL, LDH 367 U/L, TSH 5.425, T3 1.55, T4 0.77, A1c 6.5. The patient was started on levothyroxine for newly diagnosed hypothyroidism and sliding scale insulin for newly diagnosed diabetes mellitus type 2. Notably, the patient had decreased complement C3 and C4 levels, negative Anti-Glomerular Basement Membrane antibody, negative Anti-streptolysin O antibody, positive Rheumatoid Factor, positive ANCA assay, positive Proteinase antibody, mildly positive Myeloperoxidase antibody, and positive Cytoplasmic C-ANCA (Table 3).

**Table 3:** Autoimmune serology.

Study	Value	Normal Values
C3, mg/dL	56.9	82.0–185.0
C4, mg/dL	6.3	15.0–53.0
Anti-Glomerular Basement Membrane antibody	5	0–20 U
Anti-DNA antibody, double stranded	negative	negative
Antinuclear antibody (ANA)	negative	negative
Anti-streptolysin O antibody, IU/mL	86	<199
Rheumatoid Factor, IU/mL	68	<29
Jo-1 antibody, AI	<0.2	0.0–0.9
Anti-RNP, AI	0.7	0.0–0.9
Scleroderma antibody, AI	<0.2	0.0–0.9
SSA antibody, AI	<0.2	0.0–0.9
SSB antibody, AI	<0.3	0.0–0.9
Anti-Smith antibody, AI	<0.4	0.0–0.9
ANCA Assay	positive	negative
Proteinase antibody, U/mL	>100	0.0–3.5
Myeloperoxidase antibody, U/mL	24.1	0.0–9.0
Cytoplasmic C-ANCA	1:320	<1:20
Perinuclear P-ANCA	<1:20	<1:20

**Fig. 3:**
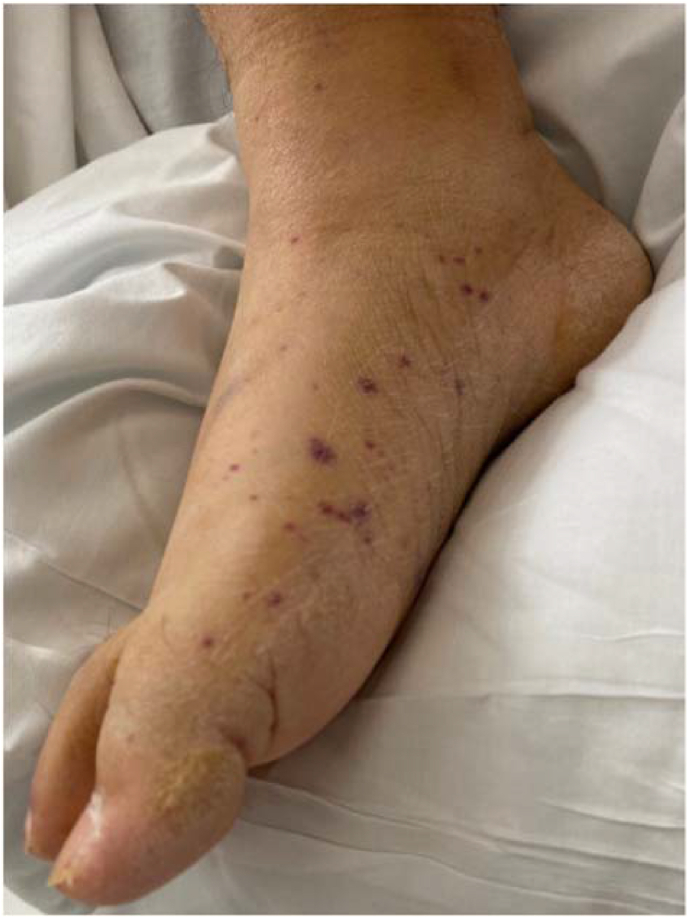
Petechial skin changes on lower extremities. Scattered, non-blanching, hemorrhagic macules on the bilateral lower extremities.

Despite hemodialysis, the patient experienced worsening AKI, ongoing hemoptysis containing clots with hypoxemia necessitating increased FiO2 requirement to 50%, with development of new onset nonpruritic petechiae that began on hospital day#3 in the bilateral lower extremities. The petechiae were associated with tenderness on the plantar surface of the foot in the calcaneal region with concomitant platelet drop from 226 to 187 (Fig. 3).

**Fig. 4:**
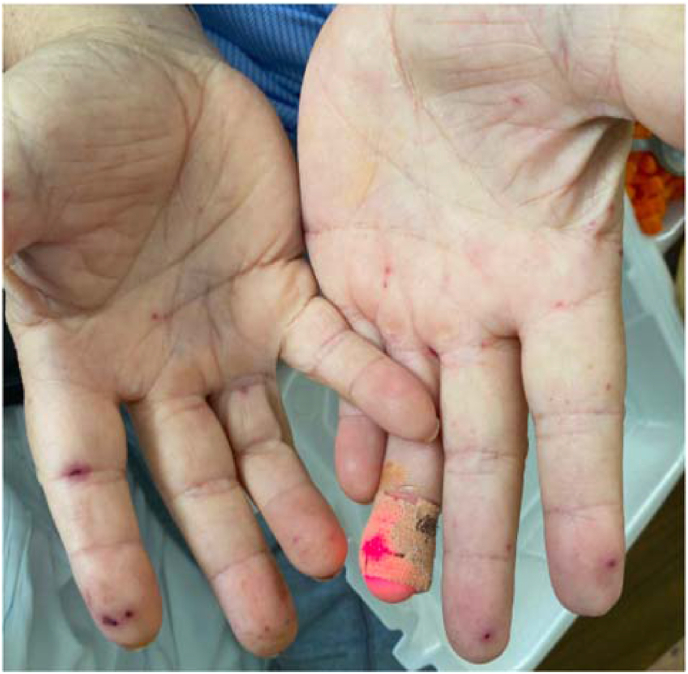
Petechial skin changes on upper extremities. Scattered, non-blanching, hemorrhagic macules on the hands.

The patient continued to develop petechial lesions in the acral regions, specifically bilateral hands, and his nose (Fig. 4).

**Fig. 5:**
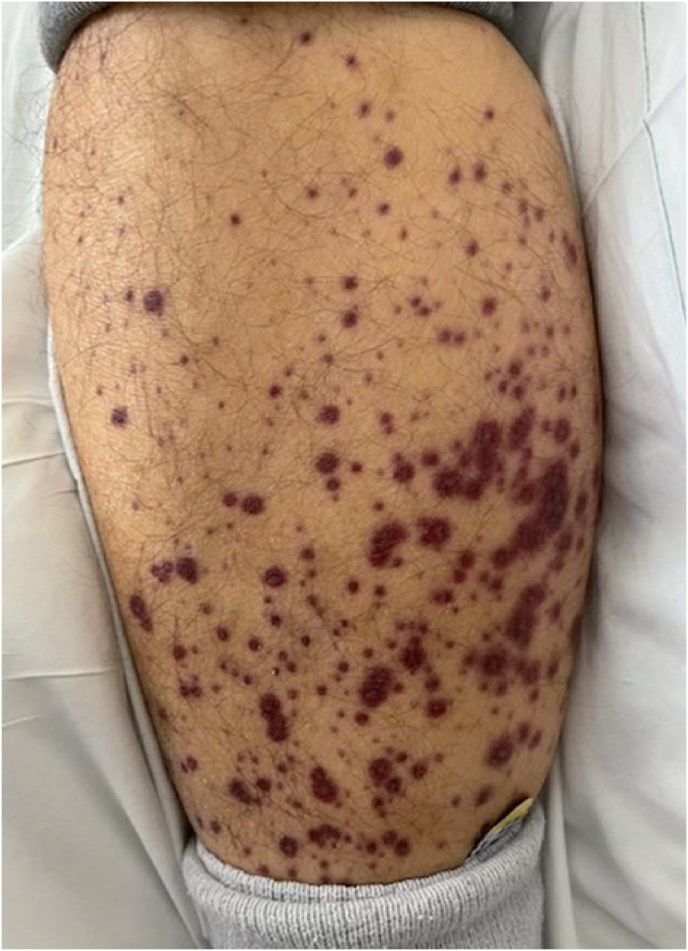
Purpuric skin manifestations. Diffuse, non-blanching, palpable, hemorrhagic papules on the bilateral lower extremities.

Previously petechial lesions on the bilateral lower extremities progressed to palpable purpura (Fig. 5).

The patient was started on an IV pulse dose of Methylprednisolone therapy 125mg every 6 hours and was also scheduled for a renal biopsy to obtain a definitive diagnosis, but this was delayed due to the increased international normalized ratio. During hemodialysis on day#5, the patient experienced increasing shortness of breath and tachycardia which prompted Computed Tomography Angiography of the chest that revealed markedly increased pulmonary infiltrates. Due to increasing respiratory distress and impending respiratory failure, the decision was made to intubate the patient, upon which active frank red bleeding arising from the trachea was noted. Shortly after intubation, the patient continued to have what looked like diffuse alveolar hemorrhage and sustained 2 consecutive PEA cardiac arrests; unfortunately, the patient expired.

### 2.1 Differential diagnosis

● Community-Acquired Pneumonia with post-inflammatory lung changes due to COVID-19
● Pulmonary Mycobacterium Tuberculosis Infection
● Goodpasture's disease
● Invasive Fungal disease (Aspergillosis/Coccidioidomycosis)
● ANCA-associated Vasculitis

## 3 Discussion

Inflammatory leukocytes in vessel walls with reactive damage to mural structures define the vasculitides. Bleeding and downstream tissue ischemia leading to necrosis present in the disease are caused by loss of vessel integrity and lumen compromise respectively [7]. The manifestation of vasculitis can occur as a primary process or secondary to an underlying condition. ANCA-associated vasculitis is a subcategory of vasculitis that has small vessel predominance. The pathogenesis of ANCA-associated vasculitis follows an initial insult that generates an aberrant pathogenic autoimmune response, followed by an active injury phase mediated predominantly by neutrophils, and a response to the injury which involves monocytes, macrophages, and T lymphocytes. Mild injury can result in complete resolution, while severe injury can result in scarring with residual dysfunction, including necrotizing glomerular injury and countless acute lesions occurring in multiple organs. The development of lesions is asynchronous in onset and can progress to chronic stages in only 1–2 weeks [7].

The diagnosis of ANCA-associated vasculitis is broken into its three small vessel vasculitis: granulomatosis with polyangiitis (GPA), microscopic polyangiitis (MPA), and eosinophilic granulomatosis with polyangiitis (EGPA). Our patients' presentations, disease manifestations, imaging, and laboratory results are suggestive of MPA or GPA (Box 1 and Box 2). [8].

Box 1 **Diagnostic Criteria for microscopic polyangiitis (MPA)**

● Symptoms
  ○ Rapidly progressive glomerulonephritis
  ○ Pulmonary hemorrhage
  ○ Other organ symptoms: Purpura, subcutaneous hemorrhage, gastrointestinal bleeding, and mononeuritis multiplex
● Histological Findings
  ○ Necrotizing vasculitis of arterioles, capillaries, and venules, and perivascular infiltration of inflammatory cells
● Laboratory findings
  ○ Positive MPO-ANCA
  ○ Positive CRP
  ○ Proteinuria, hematuria, elevation of BUN and serum creatinine
● Diagnosis
  ○ Definite MPA
    ■ Positive for 2 or more of the symptoms, and positive histological findings
    ■ Positive for 2 or more of the symptoms including the symptoms of rapidly progressive glomerulonephritis and pulmonary hemorrhage, and positive MPO-ANCA
  ○ Probable MPA
    ■ Positive for 3 of the symptoms
    ■ Positive for 1 of the symptoms, and positive MPO- ANCA

Alt-text: Box 1

Box 2 **Diagnostic Criteria for granulomatosis with polyangiitis (GPA)**

● Symptoms
  ○ E symptoms
    ■ Nose (purulent rhinorrhea, epistaxis, and saddle nose)
    ■ Eyes (ophthalmic pain, visual disturbance, and exophthalmia)
    ■ Ears (otalgia and otitis media)
    ■ Throat (pharyngeal ulcer, hoarseness, and laryngeal obstruction)
  ○ L symptoms
    ■ Bloody sputum, cough, and dyspnea
  ○ K symptoms
    ■ Hematuria, proteinuria, rapidly progressive renal failure, edema, and hypertension
  ○ Others due to vasculitis
    ■ General symptoms: fever (38 °C or higher, 2 weeks or longer), weight loss (6kg or more for 6 months)
    ■ Local symptoms: purpura, polyarthritis/polyarthralgia, episcleritis, mononeuritis multiplex, ischemic heart disease, gastrointestinal bleeding, and pleuritis
● Histological Findings
  ○ Necrotizing granulomatous vasculitis with giant cells at the sites of E, L, and/or K
  ○ Necrotizing crescentic glomerulonephritis without immune deposits
  ○ Necrotizing granulomatous vasculitis of arterioles, capillaries, and venules
● Laboratory findings
  ○ PositivePR3-ANCA (or C-ANCA by an indirect immunofluorescence)
● Diagnosis
  ○ Definite GPA
    ■ Positive for 3 or more of the symptoms, including E, L, and K symptoms
    ■ Positive for 2 or more of the symptoms, and positive for either of the histological findings
    ■ Positive for 1 or more of the symptoms, positive for either of the histological findings, and positive PR3-ANCA/C-ANCA
  ○ Probable GPA
    ■ Positive for 2 or more of the symptoms
    ■ Positive for 1 of the symptoms, and positive for either of the histological findings
    ■ Positive for 1 of the symptoms, and positive PR3-ANCA/C-ANCA

Alt-text: Box 2

The patient met probable MPA criteria with positive MPO-ANCA and symptoms. The patient met GPA criteria with positive PR3-ANCA and symptoms. Furthermore, attempts to perform a bronchoscopy in the operating were initiated multiple times during the patient's hospital course. Due to hospital protocol in the setting of Sars-CoV-2 infection, the endoscopic suites were designated for only emergency gastroenterology procedures including bronchoscopy to rule out infectious causes of DAH [9]. Due to the patient's unstable condition, kidney biopsy and confirmatory bedside bronchoscopy were not attempted. The patient expired before a definitive diagnosis could be made.

We report an initial presentation of hemoptysis leading to diffuse alveolar hemorrhage with positive C-ANCA, Myeloperoxidase antibodies, and Proteinase antibodies, suggestive of ANCA vasculitis of unknown etiology after a recent mild SARS-CoV-2 infection. The patient had a previously positive SARS-CoV-2 test and mild symptoms of COVID-19 one month prior which had resolved and was found to be negative by RT-PCR after admission. His presentation was suggestive of severe disease with diffuse alveolar hemorrhage, acute kidney injury, and purpuric cutaneous manifestations. His treatment required hemodialysis and immunosuppression. Imaging revealed bilateral patchy consolidations with cavitary lesions suggestive of multifocal pneumonia. Chest CT findings in more than 70% of RT-PCR positive SARS-CoV-2 cases describe ground-glass opacities, vascular enlargement, bilateral abnormalities, lower lobe involvement, and posterior predilection [10]. Less common CT findings describe cavitation lung lesions and could be suggestive of another diagnosis if the finding is an isolated observation [9]. COVID-19 cannot be completely ruled out from the differential in uncommon findings as these may only occur later in the disease course [10]. There is no prior evidence of ANCA-associated vasculitis developing after COVID-19 infection in a previously healthy patient nor ANCA-associated vasculitis post-COVID-19 infection with cutaneous manifestations in a previously healthy patient. A case with similar presentation was reported by Patel et al., that shared similarities in presentation and laboratory profile, however, the patient had pre-existing conditions including hypertension, hyperlipidemia, and diabetes mellitus type 2 [11].

Only hypotheses can be made when trying to find a specific trigger for ANCA-associated vasculitis in a previously healthy patient with recent COVID-19 infection, but given the pathogenesis of ANCA-associated vasculitis as an aberrant immune response to an initial immunologic insult, we conclude in this specific case COVID-19 acted as the trigger for ANCA-associated vasculitis, and diffuse alveolar hemorrhage, acute kidney injury, and purpuric manifestations were a subsequent complication; a post-acute COVID-19 syndrome.

## 4 Conclusion

SARS-CoV-2 continues to be investigated for its acute pathological insults, but the long-term consequences of the virus have yet to be fully understood. With the increasing number of people who contracted the virus and had only mild to no symptoms, unfortunately, there is an expected even larger cohort of critically ill patients in the future. In this case, we present an otherwise healthy male who contracted COVID-19 and had minimal respiratory symptoms, who then developed new-onset severe ANCA-associated vasculitis. The patient developed petechiae, acute renal failure requiring emergent hemodialysis, and severe hemoptysis which eventually led to acute respiratory failure and death. While the acute phase changes such as pulmonary inflammation and hypercoagulability are well-documented complications, the risk of developing other long-term unknown sequelae is a tragic reality that makes us wonder if the future is a possible equal threat in addition to the initial pandemic catastrophe.

Informed consent was obtained from the patient for publication of this case report and accompanying images. A copy of the written consent is available for review by the Editor-in-Chief of this journal on request.

## Author statement file

For transparency, we encourage authors to submit an author statement file outlining their individual contributions to the paper using the relevant CRediT roles: Conceptualization; Data curation; Formal analysis; Funding acquisition; Investigation; Methodology; Project administration; Resources; Software; Supervision; Validation; Visualization; Roles/Writing - original draft; Writing - review & editing. Authorship statements should be formatted with the names of authors first and credit role(s) following

Desiree Morris: Roles/writing original draft, review editing, data curation, visualization.

Kushal Patel: Roles/writing original draft, review editing, data curation.

Osman Rahimi: Data curation, roles/writing original draft.

Omar Sanyurah: Data curation, roles/writing original draft.

Alfredo Iardino: Review editing, project administration, supervision (AI and NK had same level of contribution).

Nazia Khan: Review editing, project administration, supervision (AI and NK had same level of contribution).

## Declaration of competing interest

All authors confirm that they have no conflict of interest.
